# *IKBIP* is a novel EMT-related biomarker and predicts poor survival in glioma

**DOI:** 10.1515/tnsci-2021-0002

**Published:** 2021-01-08

**Authors:** Ying Yang, Jin Wang, Shihai Xu, Wen Lv, Fei Shi, Aijun Shan

**Affiliations:** Department of Pediatrics, Futian Women and Children Health Institute, Shenzhen 518045, China; Department of Emergency, Shenzhen People’s Hospital (The Second Clinical Medical College, Jinan University; The First Affiliated Hospital, Southern University of Science and Technology), Shenzhen 518020, China

**Keywords:** glioma, *IKBIP*, epithelial-to-mesenchymal transition, prognosis

## Abstract

**Background:**

In cancer, kappa B-interacting protein (*IKBIP*) has rarely been reported. This study aimed at investigating its expression pattern and biological function in brain glioma at the transcriptional level.

**Methods:**

We selected 301 glioma patients with microarray data from CGGA database and 697 glioma patients with RNAseq data from TCGA database. Transcriptional data and clinical data of 998 samples were analyzed. Statistical analysis and figure generating were performed with R language.

**Results:**

We found that *IKBIP* expression showed positive correlation with WHO grade of glioma. *IKBIP* was increased in isocitrate dehydrogenase (IDH) wild type and mesenchymal molecular subtype of glioma. Gene ontology analysis demonstrated that *IKBIP* was profoundly associated with extracellular matrix organization, cell–substrate adhesion and response to wounding in both pan-glioma and glioblastoma. Subsequent gene set enrichment analysis revealed that *IKBIP* was particularly correlated with epithelial-to-mesenchymal transition (EMT). To further elucidate the relationship between *IKBIP* and EMT, we performed gene set variation analysis to screen the EMT-related signaling pathways and found that *IKBIP* expression was significantly associated with PI3K/AKT, hypoxia and TGF-β pathway. Moreover, *IKBIP* expression was found to be synergistic with key biomarkers of EMT, especially with N-cadherin, vimentin, snail, slug and TWIST1. Finally, higher *IKBIP* indicated significantly shorter survival for glioma patients.

**Conclusions:**

*IKBIP* was associated with more aggressive phenotypes of gliomas. Furthermore, *IKBIP* was significantly involved in EMT and could serve as an independent prognosticator in glioma.

## 1 Introduction

Gliomas account for the most common and aggressive primary brain cancers among adult patients [1]. Despite great advances in diagnosis and treatment, the prognosis for glioma patients remains unfavorable. Especially for those with glioblastoma (GBM), the most devastating type, the median survival time is only about 15 months [2,3]. Epithelial-to-mesenchymal transition (EMT) has been widely reported as a key mechanism in promoting migration, invasion and tumor progression in glioma [4]. Identification of novel EMT-related markers is of great necessity.

I kappa B kinase interacting protein (*IKBIP*), also known as *IKIP*, is on human chromosome 12. Researchers have paid little attention to this gene. Currently, we know that this gene promotes the function of apoptosis. *IKBIP* was found to be one of the target genes of *p53* and plays a crucial role in proapoptotic function [5]. Recently, *IKBIP* was identified as a vital modulator of inflammation [6]. Heretofore, the biological function of *IKBIP* in malignancies has been rarely reported. Only one study [7], through weighted gene co-expression network analysis (WGCNA), preliminarily revealed *IKBIP* as a potential hub gene in gliomagenesis. However, the role of *IKBIP* in glioma still remains largely unclear. In the present study, we took advantage of 998 glioma patients with transcriptome data to investigate the clinical significance, molecular characterization and biological function of *IKBIP* in glioma.

## 2 Materials and methods

### 2.1 Sample and data collection

Transcriptome and clinical data of glioma patients were available in Chinese Glioma Genome Atlas (CGGA) website (http://www.cgga.org.cn/) and TCGA website (http://cancergenome.nih.gov/). In total, 998 glioma patients, including 301 CGGA microarray data (GeneSpring GX 11.0 normalization) and 697 TCGA RNAseq data (RSEM normalization, level 3), were enrolled. The baseline characteristics of patients in both cohorts were described in Table S1.

First, we took advantage of CGGA microarray data to explore the *IKBIP* expression status in pan-glioma and GBM. Then TCGA RNAseq data were analyzed in parallel to further validate what we have revealed in CGGA data set and found that the consistency of results between two cohorts was fairly satisfying.

### 2.2 Statistical analysis

For TCGA cohort, RSEM RNAseq data were log2 transformed. For CGGA cohort, microarray data (already normalized and centered by data provider) were directly analyzed. Statistical analysis was performed with R language. Multiple R packages, including ggplot2, pROC [8], pheatmap, corrgram, circlize [9], gene set variation analysis (GSVA), as well as survival, were used to generate figures. The biological processes of *IKBIP*-related genes were annotated using Metascape [10] (https://metascape.org). Hallmark gene sets were downloaded from gene set enrichment analysis (GSEA) website (http://software.broadinstitute.org/) for GSEA [11] and GSVA [12]. All statistical tests were two sided, and a *p* value of <0.05 was considered statistically significant.

## 3 Results

### 3.1 *IKBIP* expression was correlated with aggressive phenotypes of glioma

**Figure 1:**
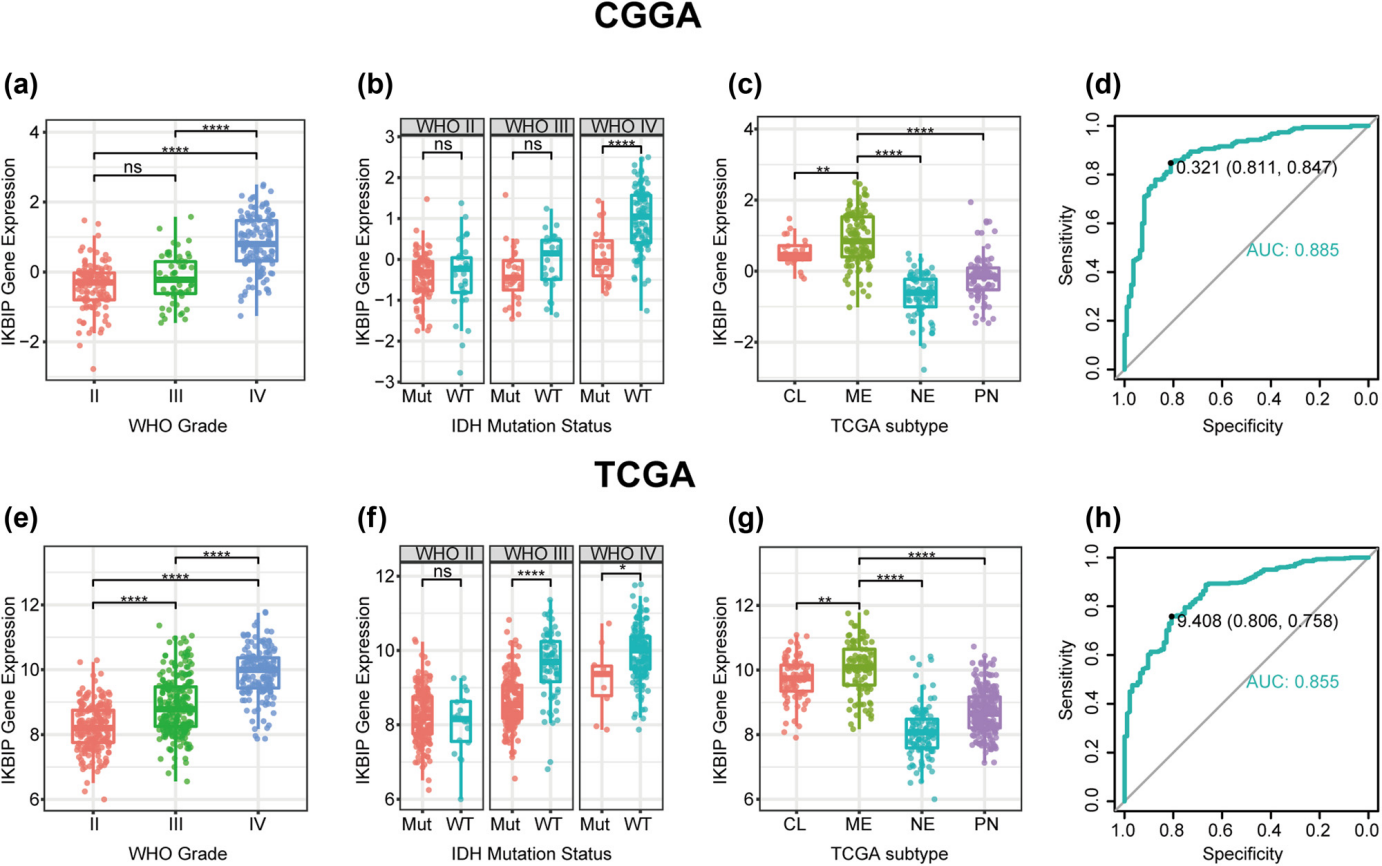
*IKBIP* expression in CGGA and TCGA data set according to the WHO grade (a and e), IDH mutation status (b and f), TCGA molecular subtype (c and g) and ROC curves (d and h) for distinguishing mesenchymal subtype. Mut, mutation; WT, wild type; CL, classical; ME, mesenchymal; NE, neural; PN, proneural. * indicates *p* value < 0.05, **indicates *p* value < 0.01, *** indicates *p* value < 0.001, **** indicates *p* value < 0.0001.

*IKBIP* expression levels were compared across different WHO grades. The results of both CGGA and TCGA cohorts consistently showed a significant positive correlation between *IKBIP* expression and WHO grade, except for comparison between WHO II and WHO III in CGGA, which also showed an apparent trend (Figure 1a and e). In addition, when patients were subclassified with respect to isocitrate dehydrogenase (IDH) mutation status, IDH wild type was found to be more associated with an increased pattern of *IKBIP* expression in both data sets, even though no statistical significance was reached in some groups (Figure 1b and f). These results suggested that higher *IKBIP* was paralleled with higher malignancy in glioma. Moreover, the correlation between *IKBIP* and TCGA molecular subtype was further examined. As shown in Figure 1c and g, *IKBIP* expression in mesenchymal subtype was significantly upregulated compared to the other subtypes, suggesting that *IKBIP* expression could contribute as a specific marker for mesenchymal subtype. The ROC curves were subsequently performed to evaluate the performance of *IKBIP* in distinguishing mesenchymal subtype. Areas under curves were 88.5% in CGGA and 85.5% in TCGA (Figure 1d and h).

### 3.2 *IKBIP*-related biological process

**Figure 2:**
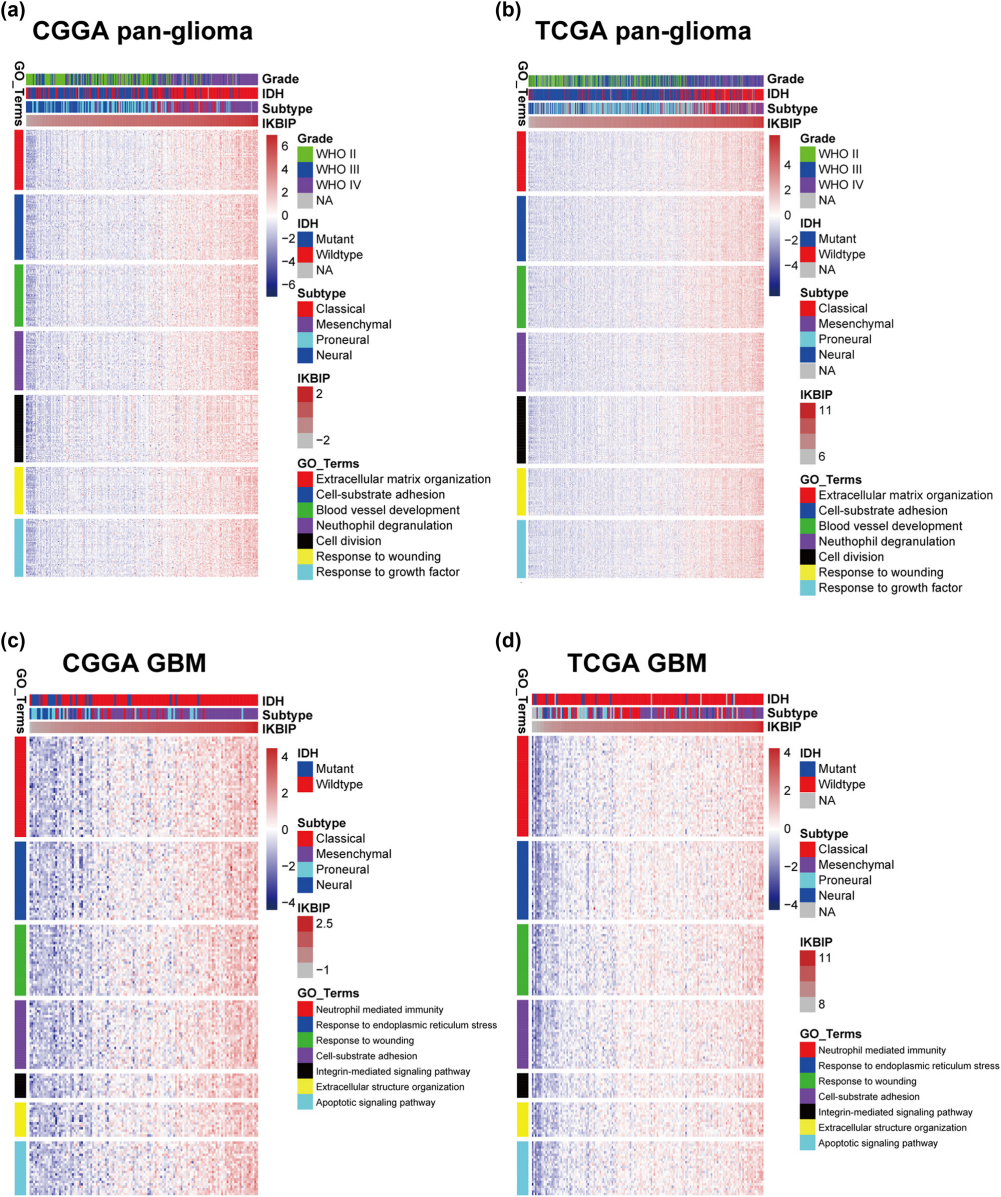
Gene ontology analysis for *IKBIP* in pan-glioma (a and b) and GBM (c and d). NA, not available.

To explore the biological process of *IKBIP* in glioma, Pearson correlation test was performed between *IKBIP* and other genes. With the criteria of Pearson coefficient |*r*| > 0.6, we identified 711 *IKBIP* positively correlated genes and 462 *IKBIP* negatively correlated genes in CGGA, and 938 *IKBIP* positively correlated genes and 17 *IKBIP* negatively correlated genes in TCGA. To ensure accuracy, *IKBIP* significantly correlated genes that were overlapped between both data sets were selected for Gene Ontology (GO) analysis. A Venn diagram (Figure S1a) was constructed, illustrating an overlap of 376 *IKBIP* positively correlated genes (Table S2), which were subsequently annotated with GO analysis. We found that *IKBIP* positively correlated genes were mainly involved in EMT-related biological processes, including extracellular structure organization, cell–substrate adhesion, blood vessel development, response to wounding, and response to growth factor. Other biological processes included neutrophil degranulation and cell division, pointing toward an association between *IKBIP* and regulation of immune response and cell cycle, respectively (Figure 2a and b).

In view of GBM as a distinct subgroup of glioma, we then conducted an independent GO enrichment analysis in this group. In GBM of both data sets, Venn diagram (Figure S1b) exhibited an overlap of 191 *IKBIP* positively correlated genes (Table S3). Subsequent GO analysis revealed that these genes were also significantly associated with EMT-related biological processes, including response to endoplasmic reticulum stress [13], response to wounding, cell–substrate adhesion, integrin-mediated signaling pathway [14] and extracellular structure organization. Besides, *IKBIP* seemed to be more associated with neutrophil-mediated immunity, suggesting that *IKBIP* upregulation was accompanied by immunosuppression of GBM, which indicated a more malignant characteristic in glioma. While it should be noted that *IKBIP* showed positive correlation with apoptotic-signaling pathway, enlightening us that *IKBIP* might also act as a proapoptotic factor in GBM [5] (Figure 2c and d).

### 3.3 *IKBIP* was associated with EMT

**Figure 3:**
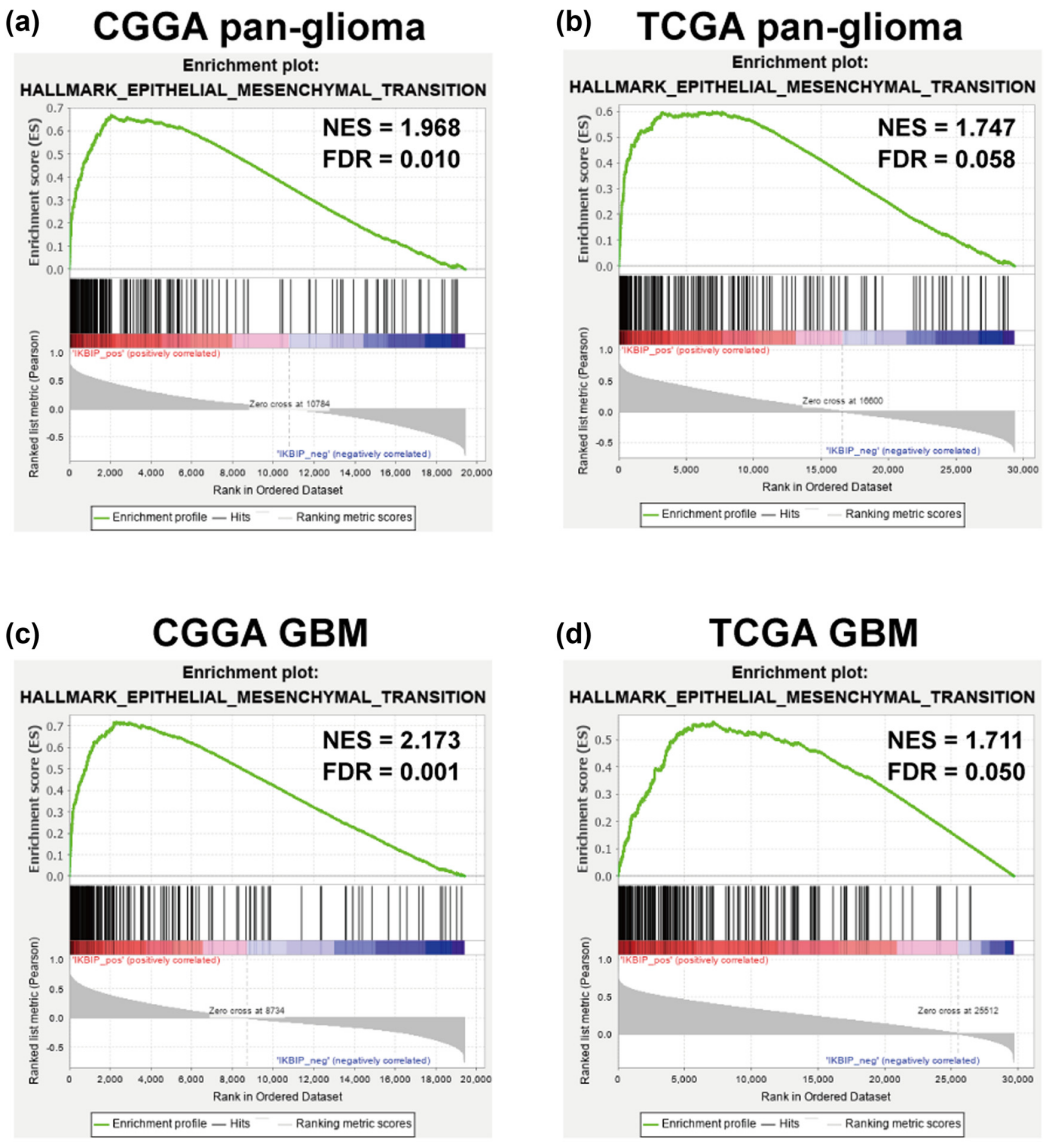
GSEA for enrichment of EMT according to *IKBIP* expression in pan-glioma (a and b) and GBM (c and d). NES, normalized enrichment score; FDR, false discovery rate.

GSEAs were performed in both CGGA and TCGA data sets, and it turned out that *IKBIP* was significantly correlated with EMT in CGGA (normalized enrichment score (NES) = 1.968, false discovery rate (FDR) = 0.010; Figure 3a), which was further validated in TCGA (NES = 1.747, FDR = 0.058; Figure 3b). Furthermore, in GBM, *IKBIP* showed an even higher association with EMT in both cohorts (Figure 3c and d). These results indicated that *IKBIP* could be profoundly associated with EMT phenotype in glioma.

### 3.4 *IKBIP* interacted with EMT-related signaling pathways in glioma

**Figure 4:**
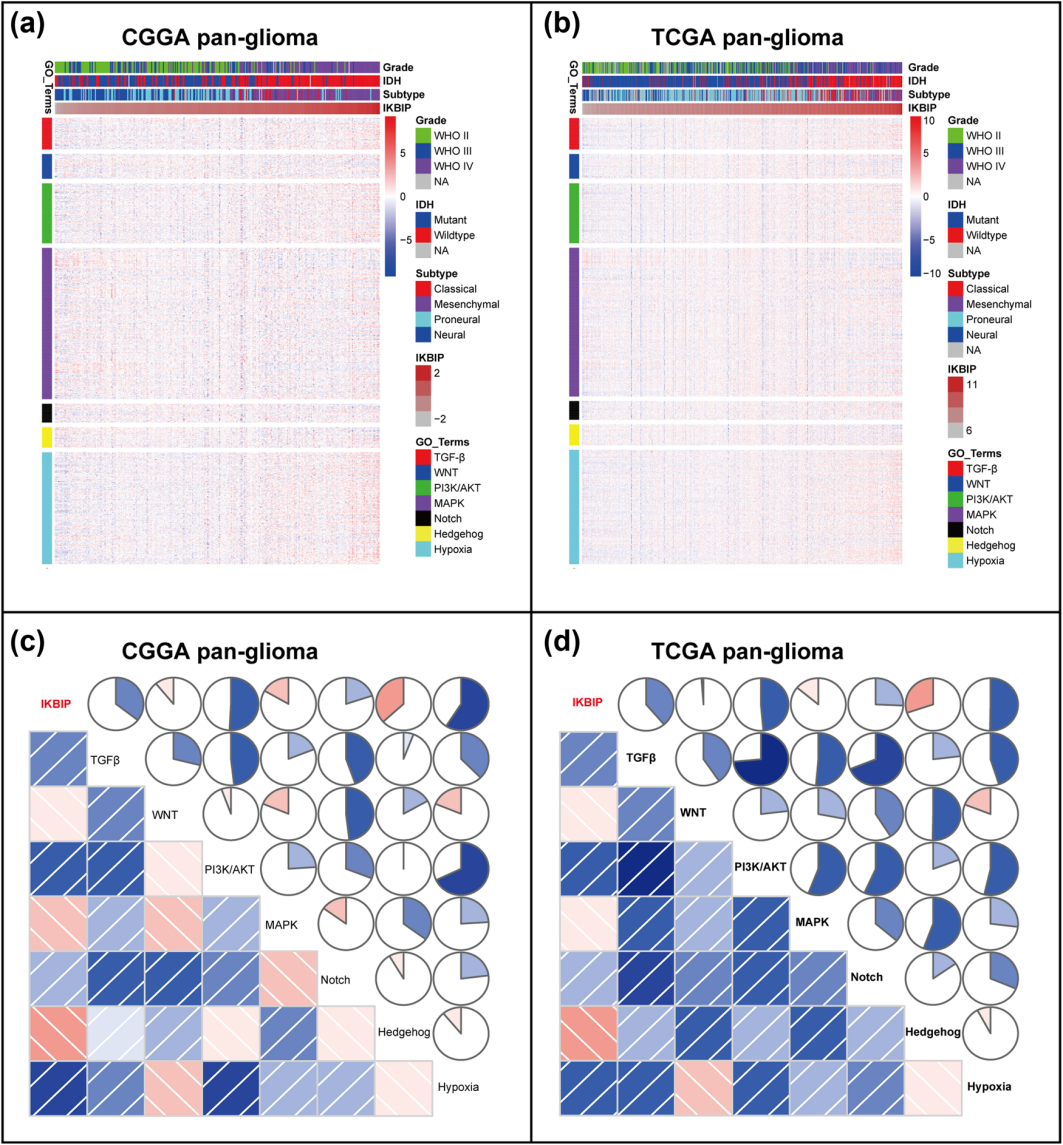
Cluster (a and b) and GSVA (c and d) of *IKBIP*-related EMT-signaling pathways in pan-glioma. NA, not available.

**Figure 5:**
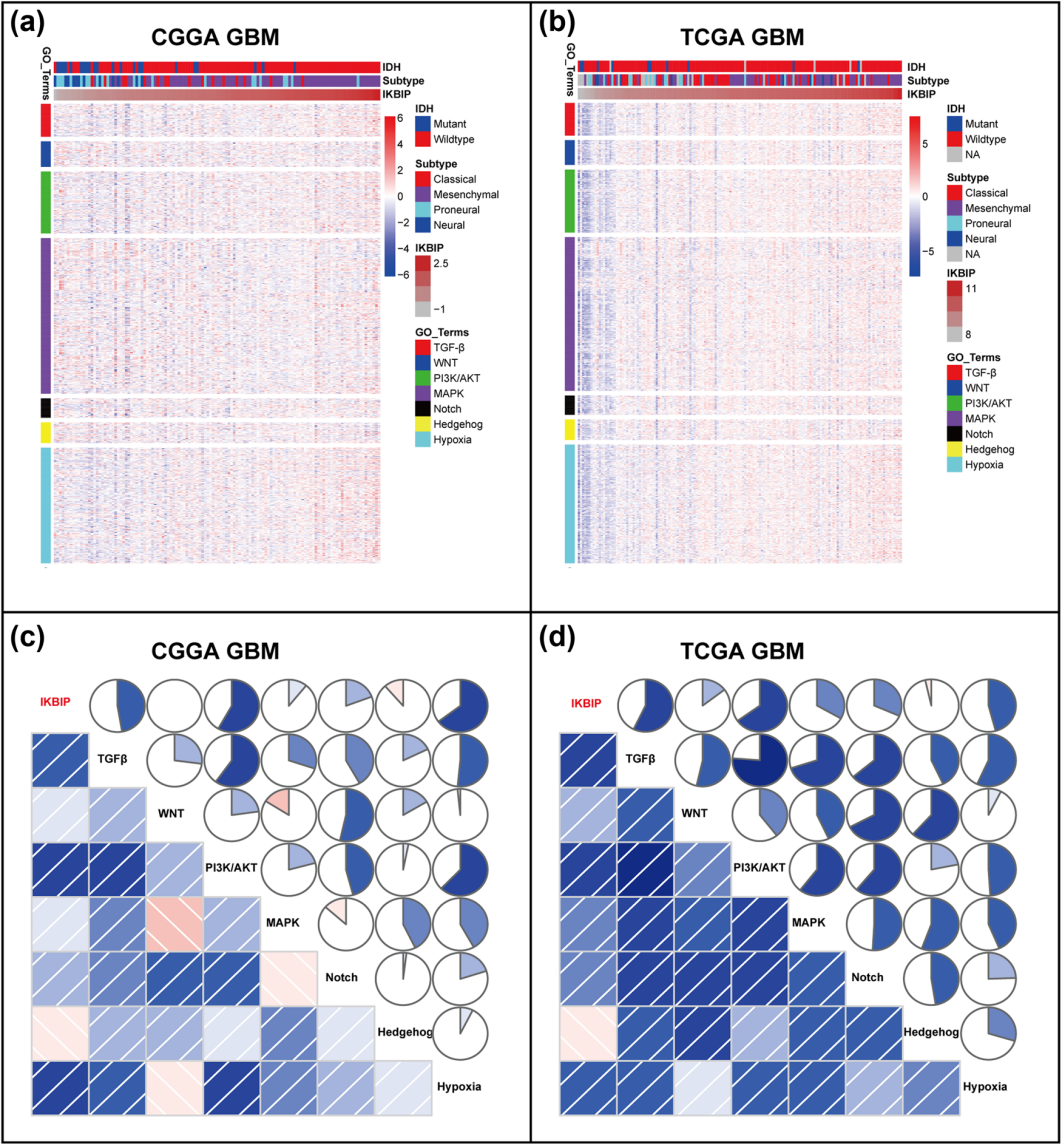
Cluster (a and b) and GSVA (c and d) of *IKBIP*-related EMT-signaling pathways in GBM. NA, not available.

To further investigate the relationship between *IKBIP* and EMT, we downloaded seven gene sets from GSEA website (Table S4), which were subsequently transformed into metagenes, representing different EMT-related signaling pathways, summarized by Gonzalez et al. [15]. As shown in Figure 4a and b, three clusters, including TGF-β-, PI3K/AKT-, and hypoxia-signaling pathways, were significantly associated with *IKBIP* expression. To quantify what we observed in clusters, GSVA was performed to generate seven metagenes based on the corresponding genes of seven EMT-related signaling pathways. According to the Pearson *r* value between *IKBIP* and seven metagenes, Corrgrams were generated to evaluate their interrelations (Figure 4c and d). *IKBIP* showed a robust correlation with TGF-β, PI3K/AKT and hypoxia signaling pathway, while only showed a weak correlation with WNT, MAPK, NOTCH and HEDGEHOG pathway, in consistent with what we observed in Figure 4a and b. Moreover, a similar pattern of EMT-related signaling pathways was observed in GBM of both CGGA and TCGA data set (Figure 5).

### 3.5 *IKBIP* interacted with EMT-related key biomarkers in glioma

**Figure 6:**
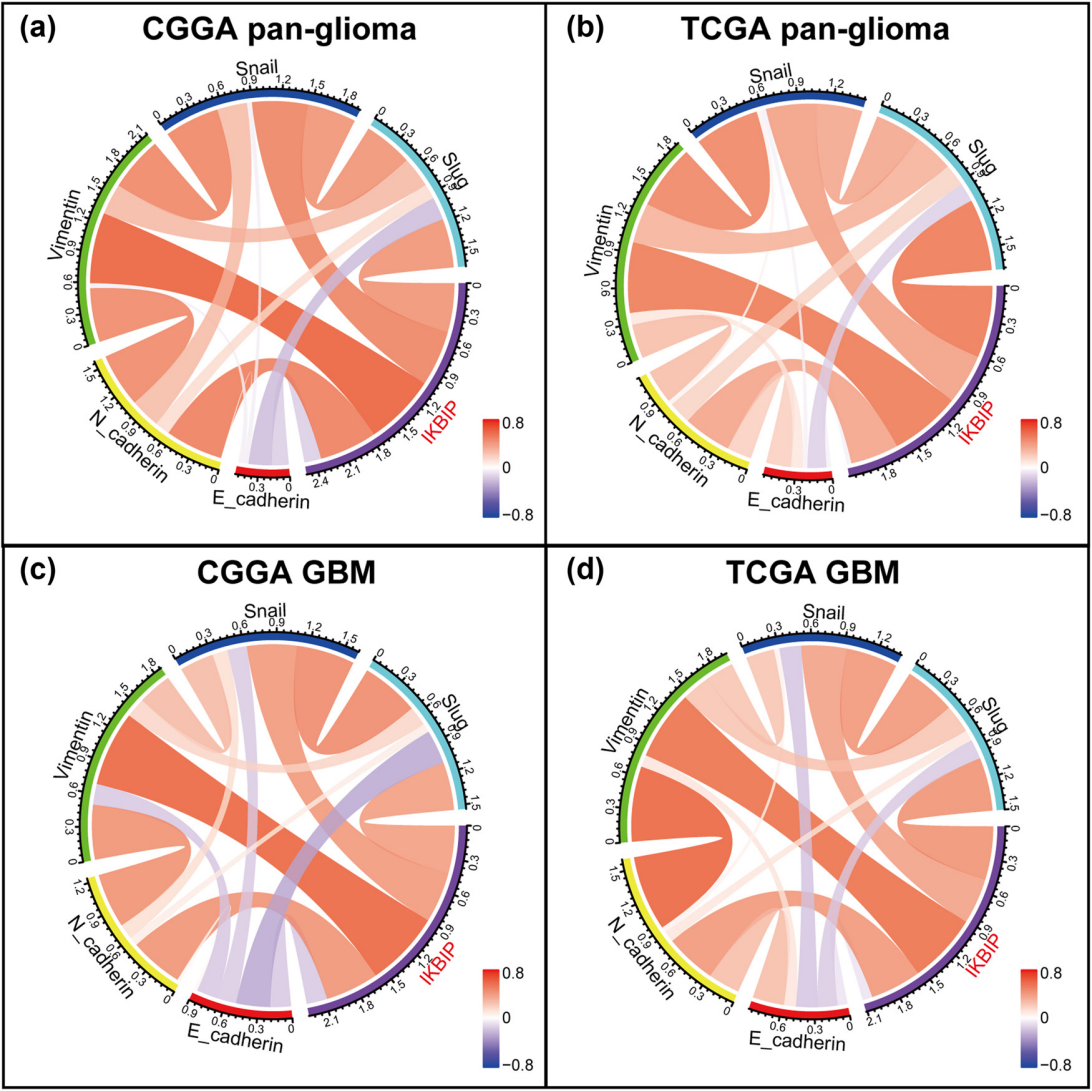
Correlation of *IKBIP* and EMT key biomarkers.

To further validate the role of *IKBIP* in EMT-related signaling pathways, we examined the correlation between *IKBIP* and EMT-related key biomarkers, including *E-cadherin*, *N-cadherin*, *vimentin*, *snail* and *slug*. Circos plots revealed that *IKBIP* expression was significantly associated with *N-cadherin*, *vimentin*, *snail* and *slug* (Figure 6a and b). To further demonstrate the interaction of these markers in GBM, Pearson correlation tests were additionally performed. As shown in Figure 6c and d, the correlation between *IKBIP* and these markers in GBM was also very robust in both data sets, indicating synergistic effects of these members during glioma EMT. The correlation between *IKBIP* and *E-cadherin* was very weak, and this might be deemed as a noise.

**Figure 7:**
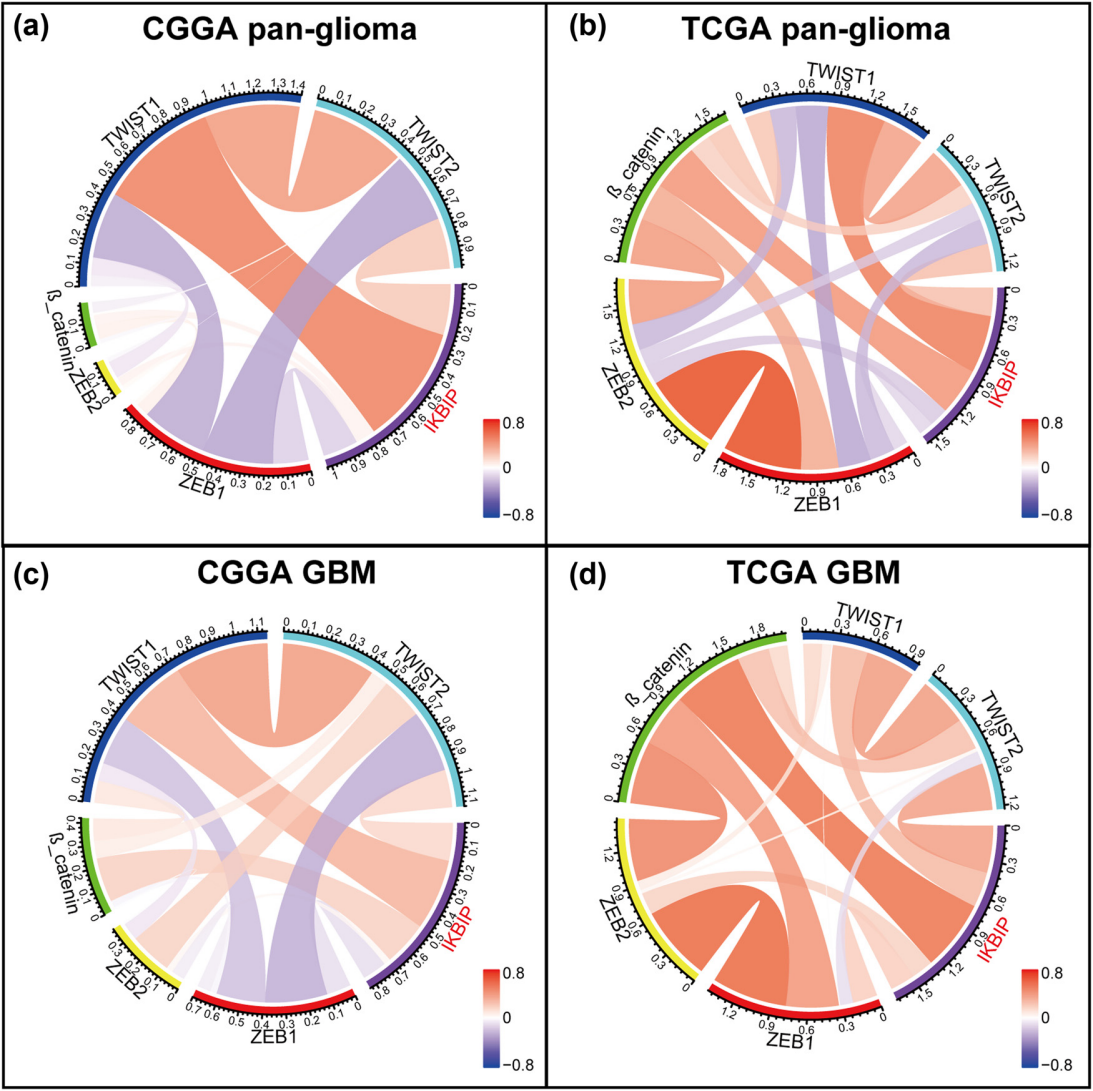
Correlation of *IKBIP* and other EMT key biomarkers.

In EMT, many other molecules have been identified as EMT-related key biomarkers [16]. We additionally enrolled EMT-related markers including *ZEB1/2*, *β-catenin* and *TWIST1/2* and put them into analysis together with *IKBIP*. Subsequent Circos plots in both CGGA and TCGA congruently revealed that *IKBIP* expression was especially correlated with *TWIST1* in both pan-glioma and GBM (Figure 7).

### 3.6 Higher *IKBIP* predicts shorter survival for glioma

**Figure 8:**
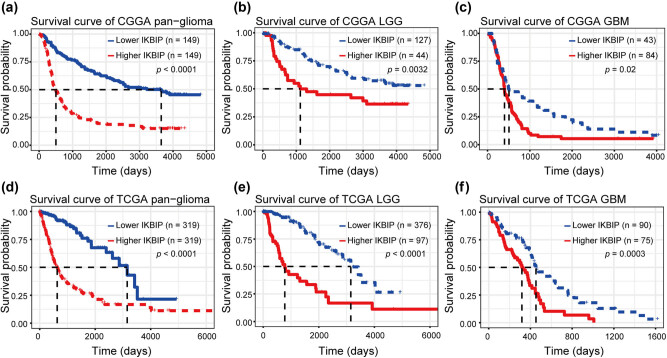
Survival analysis for *IKBIP* in pan-glioma (a and d), LGG (b and e) and GBM (c and f).

Kaplan–Meier (KM) survival analyses were performed to examine the prognostic value of *IKBIP* in glioma. According to *IKBIP* expression, pan-glioma samples were divided into two groups in each data set. As shown in Figure 8a and d, a higher level of *IKBIP* expression predicted a significantly shorter survival. Moreover, a similar pattern of the KM survival curve was observed among patients with lower grade glioma (LGG) (Figure 8b and e) and GBM (Figure 8c and f).

## 4 Discussion

In the present study, we investigated the transcriptional expression profiles of *IKBIP* in 998 glioma patients and revealed that *IKBIP* expression showed significant positive correlation with the WHO grade of glioma. Furthermore, higher *IKBIP* expression was usually accompanied by a more aggressive and malignant phenotype in glioma, including GBM, IDH wild type and mesenchymal subtype. Moreover, higher *IKBIP* expression indicated a significantly shorter survival for patients with glioma, across different WHO grades. These findings suggested that *IKBIP* played a vital role in the malignant progression of gliomas, in line with the results of a previous WGCNA study [7]. Understanding the molecular mechanism of *IKBIP* in glioma may provide a novel therapeutic target to overcome this fatal disease.

To elucidate the biological function of *IKBIP* in glioma, GO analysis was performed, and it turned out that *IKBIP* was highly associated with a series of EMT-related biological processes, including extracellular matrix organization, cell–substrate adhesion and response to wounding in both pan-glioma and GBM. Subsequent GSEA analysis revealed remarkable evidence because *IKBIP* was particularly correlated with EMT, which had been extensively confirmed to play a key role not only in glioma migration/invasion but also in glioma recurrence and therapeutic resistance [17,18,19]. These results enlightened us that *IKBIP* might promote tumorigenesis and progression of glioma mainly by means of EMT induction, which has yet been previously reported. Besides, GO analysis also revealed that *IKBIP* played a crucial role in tumor-induced immune and inflammatory response in glioma, especially in GBM, in line with the results presented by Wu et al. [6]. They demonstrated that *IKBIP* played an inhibitory role in immune and inflammatory response through negative regulation of NF-κB pathway. Based on these, we concluded that apart from being a key molecule for EMT induction, *IKBIP* might contribute as an immune suppressor in glioma as well, which further validated its oncogenic role in glioma. Meanwhile, it was noteworthy that *IKBIP* showed robust correlation with apoptotic-signaling pathway in GBM, suggesting a potential proapoptotic function [5]. As a result, we speculated that *IKBIP* might have a dualistic nature in gliomagenesis, and the robust protumoral effect through EMT induction and immune inhibition overwhelmed the antitumoral effect through proapoptotic function.

To further validate the pro-EMT effect of *IKBIP* in glioma, we selected a series of EMT-related signaling pathways and biomarkers, which were then analyzed to determine their interaction with *IKBIP* and found that *IKBIP* showed robust correlation with PI3K/AKT-, hypoxia- and TGF-β-signaling pathway, suggesting that *IKBIP* might promote EMT process through these pathways. Moreover, most of EMT biomarkers, including *N-cadherin*, *snail*, *slug*, *vimentin* and *TWIST1* were significantly associated with *IKBIP*, indicating that *IKBIP* interacted synergistically with these key molecules of EMT. These results further validated the involvement of *IKBIP* in glioma EMT. Thus, our findings might bring a novel EMT target for potential glioma treatment.

In conclusion, *IKBIP* expression was associated with more aggressive phenotypes of glioma and predicted much worse survival for patients. Moreover, *IKBIP* was significantly associated with EMT process and interacted synergistically with EMT-related signaling pathways and key biomarkers. However, a limitation of the current study was that no experimental validation was performed. Further *in vitro* and *in vivo* studies are needed to validate its role in glioma.
